# Acute stress increases left hemispheric activity measured via changes in frontal alpha asymmetries

**DOI:** 10.1016/j.isci.2022.103841

**Published:** 2022-02-01

**Authors:** Gesa Berretz, Julian Packheiser, Oliver T. Wolf, Sebastian Ocklenburg

**Affiliations:** 1 Department of Biopsychology, Institute of Cognitive Neuroscience, Faculty of Psychology, Ruhr University Bochum, Universitätsstraße 150, Room: IB 6/109, 44780 Bochum, Germany; 2 Netherlands Institute for Neuroscience, Social Brain Lab, Amsterdam, the Netherlands; 3 Department of Cognitive Psychology, Institute of Cognitive Neuroscience, Faculty of Psychology, Ruhr University Bochum, Bochum, Germany; 4 Department of Psychology, Medical School Hamburg, Hamburg, Germany; 5 Institute for Cognitive and Affective Neuroscience, Medical School Hamburg, Hamburg, Germany

**Keywords:** Health sciences, Electrodiagnostic medicine, Biological sciences, Neuroscience, Clinical neuroscience

## Abstract

Frontal EEG alpha band asymmetries have been linked to affective processing in healthy individuals and affective disorders. As stress provides a strong source of negative affect, the present study investigated how acute stress affects frontal EEG alpha asymmetries. Continuous EEG data were acquired from 51 healthy adult participants during stress induction with the Trier Social Stress Test. EEG data were also collected during a non-stressful control condition. Furthermore, EEG resting state data were acquired after both conditions. Under stress, participants showed stronger left hemispheric activation over frontal electrodes as well as reduced left-hemispheric activation over occipital electrodes compared to the control condition. Our results are in line with predictions of the asymmetric inhibition model which postulates that the left prefrontal cortex inhibits negative distractors. Moreover, the results support the capability model of emotional regulation which states that frontal asymmetries during emotional challenge are more pronounced compared to asymmetries during rest.

## Highlights

• EEG recording during social stress induction
• Stronger left hemispheric frontal activation during emotional challenge
• No stress-related changes in resting state EEG after stress induction
• Support for asymmetric inhibition and the capability model of emotional regulation

## Graphical abstract

**Figure:**
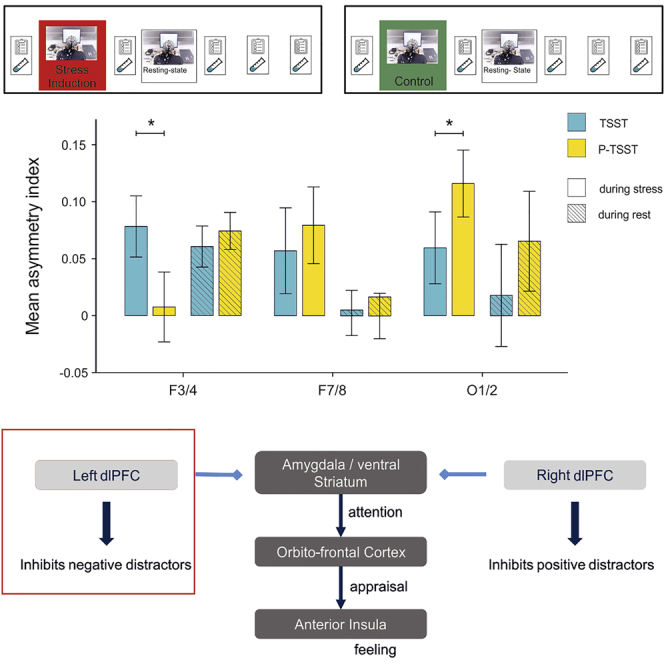

## Introduction

Although the healthy brain exhibits asymmetries on both the structural and the functional level (Kong et al., 2018), a number of mental disorders have been associated with changes in these asymmetries (Berretz et al., 2020). Although recent research has focused on changes in structural asymmetries (Kong et al., 2020; Postema et al., 2019), changes in functional asymmetry have been linked to symptoms in these disorders as well (Ocklenburg et al., 2015). For example, patients suffering from posttraumatic stress disorder (PTSD) demonstrate right-sided hyperactivity in frontal areas (Meyer et al., 2015) with changes in asymmetry in response to trauma cues distinguishing patients from healthy controls (Meyer et al., 2018). In major depressive disorder (MDD), patients typically display reduced left frontal or increased right frontal alpha band activity in the EEG (Lopez-Duran et al., 2012). These changes in asymmetry seem to be frequent in patients diagnosed with MDD (Thibodeau et al., 2006). However, they seem to be insufficient as a diagnostic marker (van der Vinne et al., 2017). Many of these mental disorders are also associated with changes in emotional processing (Lizeretti et al., 2012). Asymmetries in alpha band frequency power over frontal electrodes have been associated with trait and state dependent affective processing (Reznik and Allen, 2018). Alpha power has been proposed to reflect functional inhibition in an area, meaning that higher alpha power is associated with lower activation of that area (Jensen and Mazaheri, 2010).

More right-sided alpha power indicating more left-hemispheric activation (Allen et al., 2004) has been linked to decreased negative affect (Tomarken et al., 1992), increased emotional flexibility (Papousek et al., 2012), and increased emotional regulation (Hannesdóttir et al., 2010). Moreover, decreased left frontal activity can be used as a predictor for anxiety under social threat (Crost et al., 2008). Social threat represents a potent influence on the nervous system reliably leading to the subjective feeling of stress as well as a strong acute hormonal stress response (Dickerson and Kemeny, 2004). The latter can be subdivided into two different stress response systems. Activation of the sympathetic nervous system leads to a release of epinephrine and norepinephrine from the adrenal medulla. The slower hypothalamus-pituitary-adrenocortical (HPA) axis results in the production of cortisol which in turn can exert its effects on the brain and body (Joëls and Baram, 2009).

Interestingly, many disorders display not only changes in asymmetries but also alterations in basal cortisol levels and HPA reactivity (Berretz et al., 2020). Research thus far has focused on the association between altered asymmetries and early life and chronic stress as for example children with suboptimal intrauterine environments indicated by lower birth weights display higher rates of left-handedness (de Kovel et al., 2019). For example, Mundorf et al. (2020) could show in a recent study that prolonged stress exposure in early life led to an induction of atypical asymmetric behavior in rats.

The number of studies investigating the association between acute stress and asymmetries are sparse, however. Even though there seems to be an apparent link (Berretz et al., 2020), to our knowledge, only two studies investigated the role of frontal alpha asymmetries after acute stress induction. Zhang et al. (2018) found a shift toward greater right frontal activation during a cold pressor test, where participants have to submerge their feet in cold water eliciting a stress response. However, this change was not correlated with physiological or subjective stress responses. The other study was performed by Quaedflieg et al. (2015) who measured frontal alpha asymmetries before and after stress induction via the Maastricht Acute Stress Test (Smeets et al., 2012). The authors found that left frontal activity at baseline 15 min before stress was associated with smaller cortisol responses to acute stress. They hypothesized that frontal alpha asymmetry at baseline acts as a moderator in the acute stress response to downregulate the neuroendocrine reaction to stress.

As this research has demonstrated, acute stress possesses the capacity to modulate frontal alpha asymmetries. However, the previous studies have focused on changes during rest after stress induction rather than during stress exposure itself. According to the capability model of individual differences in frontal alpha asymmetry (Coan et al., 2006), frontal asymmetries during emotional challenge are more pronounced compared to asymmetries during rest conditions as they reflect the individual's capability for emotional regulation. However, to our knowledge, no study has investigated changes in frontal alpha asymmetries during acute social stress induction. To fill this gap, we recorded continuous EEG measures during performance of the Trier Social Stress Test (TSST; Kirschbaum et al., 1993) and a placebo/control condition (P-TSST; Het et al., 2009). We focused on changes in frontal alpha asymmetries in response to stress at the F3/4 and F7/8 electrode pairs that have been implicated in previous research (Düsing et al., 2016; Quaedflieg et al., 2015). To put these possible effects into context of the stress reaction, we also investigated alpha asymmetries at the O1/2 electrode pair, as a control condition. This was done because the visual cortex was found to generate highly coherent alpha oscillations (Cantero et al., 2002) but is unlikely to be involved in stress processing (Berretz et al., 2021). To test the predictions of the capability model, we also collected resting state EEG after acute stress induction and the control procedure. We hypothesize stronger alpha asymmetries during acute stress induction in the stress compared to the following resting state recording.

## Results

### Stress manipulation

**Figure 1:**
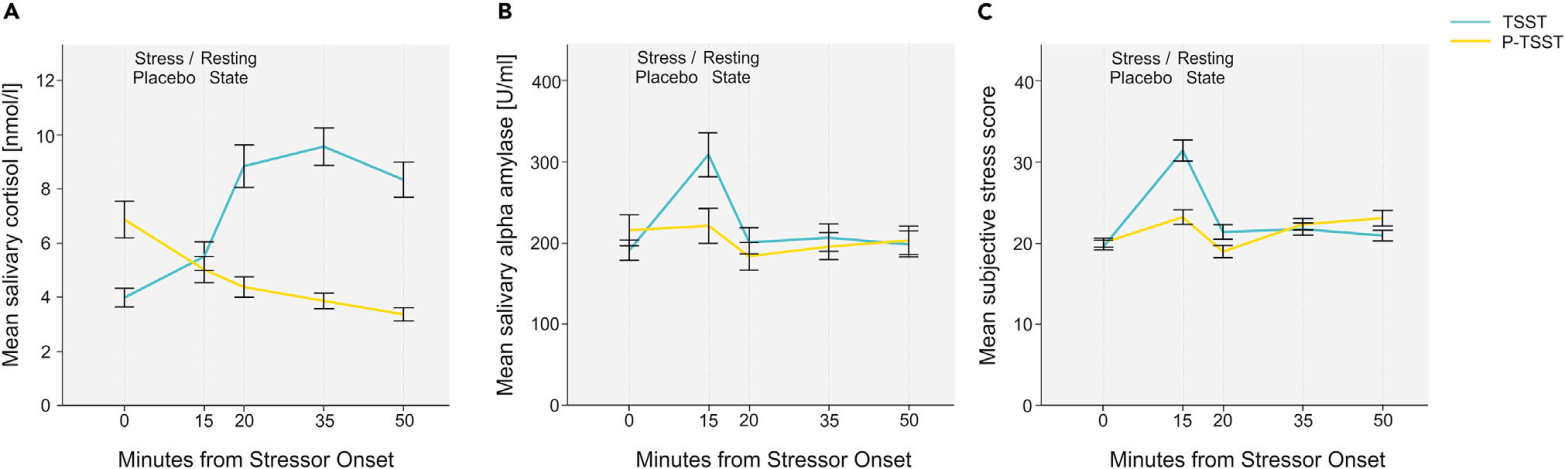
Physiological and subjective stress reactions in the TSST and P-TSST sessions Error bars represent 1 ± SE of the mean (SEM). The first measurement was taken immediately before the TSST or P-TSST preparation period. (A) Mean log-transformed cortisol in relation to measurement time point. (B) Mean salivary alpha amylase response in relation to measurement time point. (C) Mean subjective stress responses measured by SERS in relation to measurement time point.

To determine the efficacy of our stress induction, we performed a 2 × 5 repeated measures ANOVA with the factors condition (TSST, P-TSST) and time point of measurement (1–5) for cortisol, salivary alpha amylase, and affect. For cortisol (see Figure 1A), we found a significant main effect of condition (F_(1,50)_ = 25.24, *p* <0 .001, η_p_^2^ = 0.34) and time (F_(4,200)_ = 44.87, *p* = 0.002, η_p_^2^ = 0.10). There was also a significant interaction effect of both (F_(4,200)_ = 45.43, *p* <0 .001, η_p_^2^ = 0.48) indicating that the stress induction was successful over time. To provide more detailed insights, we calculated Bonferroni-corrected post hoc tests of the factor condition. The test revealed that cortisol levels were increased in the P-TSST condition (*p* <0 .001) compared to the TSST condition at T_0_. At T_20_, T_35_, and T_50_, cortisol levels were increased in the TSST condition (*p*s<0.001) (see Table S1 for descriptive data). To test whether order of test conditions had an effect on baseline cortisol levels, we additionally performed a repeated measures mixed ANOVA with the within subject factor condition and the between subject factor of order. The analysis revealed a significant effect of condition (F_(4, 49)_ = 16.85, *p* <0 .001, η_p_^2^ = 0.26) indicating that cortisol was increased in the TSST compared to the P-TSST condition. There was no interaction effect of order (F_(4, 49)_ = 0.52, *p* = 0.475, η_p_^2^ = 0.01) suggesting that these results were not affected by the order in which participants were subjected to the conditions.

We repeated the identical analysis for salivary alpha amylase (see Figure 1B) to identify whether similar effects could be seen in the sympathetic nervous system. We found a significant main effect of time (F_(4,200)_ = 21.12 *p* <0 .001, η_p_^2^ = 0.30). Similar to the cortisol analysis, there was also a significant interaction effect of condition and time (F_(4,200)_ = 18.27, *p* <0 .001, η_p_^2^ = 0.27) suggesting that sAA levels changed at selected time points following the procedure. Bonferroni-corrected post hoc tests of the factor condition revealed that sAA levels were increased in the P-TSST condition (*p* = 0.031) compared to the TSST condition at T_0_. At T_15_, sAA levels were increased in the TSST condition compared to the P-TSST condition (*p* <0 .001) indicating that the stress induction led to an increase in sympathetic activity. However, this increase was short lasting as no difference could be detected at T_20_, T_35_, and T_50_ (all *p*s > 0.05). Again, to test whether order of conditions had an effect on baseline sAA levels, we calculated a repeated measures mixed ANOVA with the within subject factor condition and the between subject factor of order. The analysis revealed no significant effect of condition (F_(4, 49)_ = 0.74, *p* <0 .394, η_p_^2^ = 0.02). There was also no significant interaction with order (F_(4, 49)_ = 3.09, *p* = 0.085, η_p_^2^ = 0.06) suggesting that these results were not affected by the order of testing conditions.

Finally, the analysis was conducted identically for affect measurement using the SERS (see Figure 1C). We found a significant main effect of time (F_(4,200)_ = 58.44, *p* <0 .001, η_p_^2^ = 0.54) indicating that subjective stress changed over time. Similar to cortisol and sAA, there was a significant interaction effect between the factors condition and time (F_(4,200)_ = 23.98, *p* <0 .001, η_p_^2^ = 0.32). At T_15_ and T_20_, subjective stress ratings were increased in the TSST condition (*ps* <0 .041) compared to the P-TSST condition as indicated by Bonferroni-corrected post hoc tests for the factor condition. At T_50_, subjective stress levels were increased in the P-TSST condition compared to the TSST condition (*p*s<0.049). Finally, we performed repeated measures mixed ANOVA with the within subject factor condition and the between subject factor in order to test whether order of test conditions had an effect on baseline subjective stress levels. The analysis revealed no significant main effect of condition (F_(4, 49)_ = 0.08, *p* = 0.780, η_p_^2^ = 0.002) nor a significant interaction with order (F_(4, 49)_ = 1.52, *p* = 0.223, η_p_^2^ = 0.03).

### EEG during stress

In a first step, we assessed the overall data quality in the EEG signal. We calculated the number of rejected segments in the artifact rejection for the TSST and P-TSST sessions and performed a dependent sample t-test. Overall, less than 10% of segments had to be rejected in both sessions suggesting that our data were largely unaffected by artifacts (mean percent of rejected segments for the TSST: 9.03% SD = 11.82%, mean percent of rejected segments for the P-TSST: 7.23%, SD = 11.51%). There was no difference between the two test sessions (t_(50)_ = 1.23, *p* = 0.225) indicating that data quality was sufficient and comparable between them.

#### Influence of stress on asymmetry

**Figure 2:**
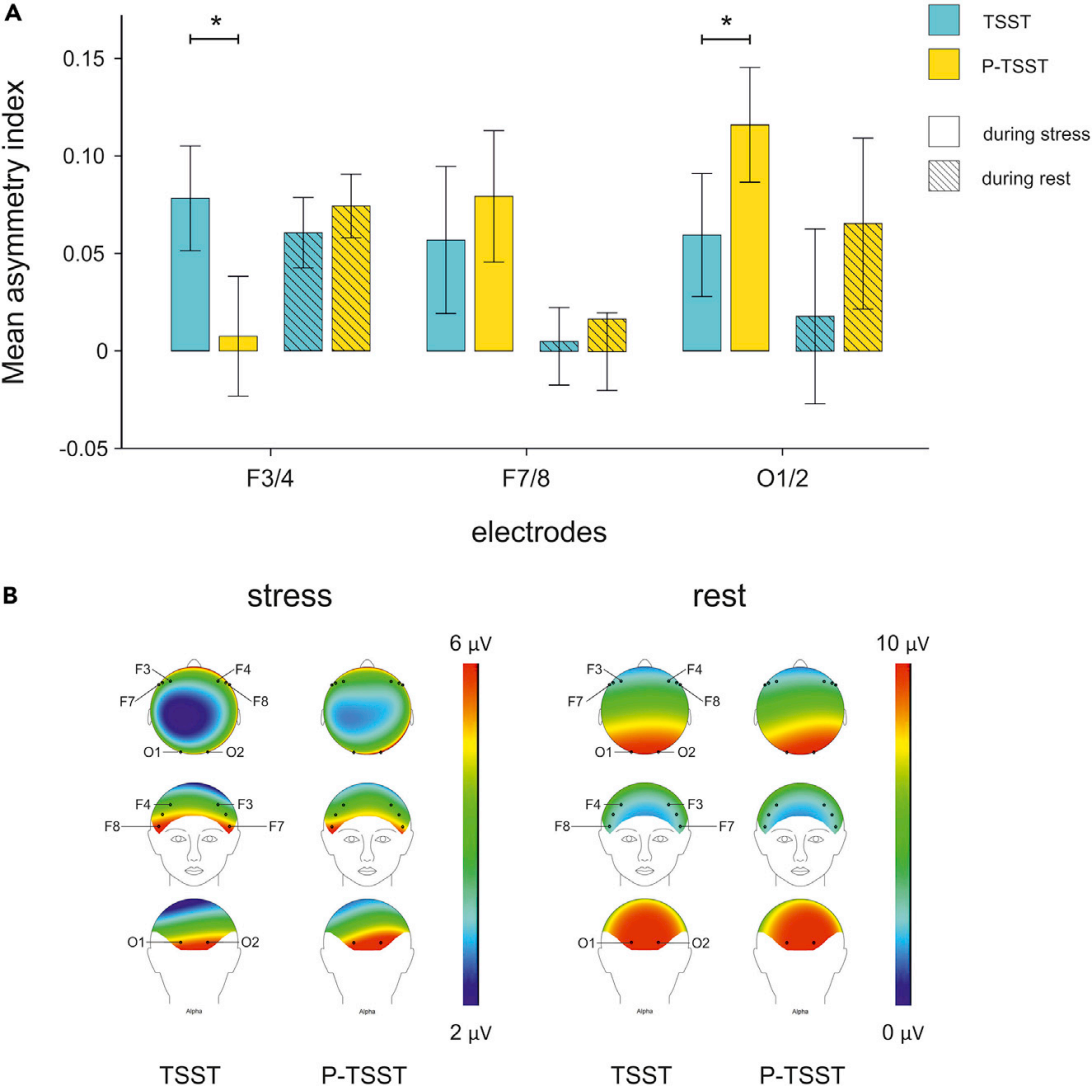
Results of the EEG recordings (A) Alpha asymmetry indices for the stress (TSST) and no stress (P-TSST) condition over frontal and occipital electrodes. Solid colors indicate AIs during stress induction, whereas hatched colors show AIs during rest. Over the F3/F4 electrode pair, we found more positive AIs in the TSST compared to the P-TSST session indicating stronger left-hemispheric activation during stress induction. On the O1/O2 electrode pair, we found the opposite effect. Error bars represent ±1 SEM ∗ reflect significant differences with *p* < 0.05. (B) Alpha power distribution across the skull. Electrodes of interest are highlighted. Please note that the frontal view is laterally reversed. During the TSST, there was stronger right frontal alpha power compared to P-TSST condition whereas we found stronger left frontal alpha power over occipital sites during stress. During resting state, there were no significant differences between the TSST and P-TSST conditions.

To investigate differences in EEG asymmetries at our electrodes of interest, we performed a 2 x 3 repeated measures ANOVA with the factors condition (TSST, P-TSST) and electrode pair (F3/4, F7/8, O1/2) for AIs in alpha frequency band power (see Figure 2, see Table S2). We found no significant main effect of either factor but a significant interaction between condition and electrode pair (F_(2,100)_ = 3.36, *p* = 0.039, η_p_^2^ = 0.06; see Table S3). To elucidate this interaction, we computed a Bonferroni-corrected post hoc test for the factor condition that revealed a significantly higher AI at the F3/4 electrode pair in the TSST session compared to the P-TSST session (*p* = 0.048). This demonstrates stronger right-hemispheric alpha power under stress which is indicative of more left-hemispheric activation. This increase was because of higher alpha power over the right hemisphere under stress (see Table S4). At the O1/2 electrode pair, this effect was reversed with lower AI scores in the TSST session (*p* = 0.028). This change was because of a decrease in alpha power over the right hemisphere suggesting stronger right-hemispheric activation under stress (see Table S4). We did not find any significant differences between the TSST and the P-TSST session for the F7/8 electrode pair (*p* = 0.617). Exploratory results for all electrode pairs can be found in Figure S2.

We calculated bivariate Pearson correlations between stress markers and AIs at the electrodes of interest. None of the correlations were significant (see Table 1).

**Table 1:** Pearson correlations coefficients between stress markers and AIs during stress

		AUCg sAA	AUCi sAA	AUCg cortisol	AUCi cortisol	AI F4/F3	AI F8/F7	AI O2/O1
AUCg sAA	R	1	0.84^b^	−0.03	−0.16	−0.13	−0.02	−0.14
	P		0.00	0.85	0.26	0.35	0.87	0.35
AUCi sAA	R	0.84^b^	1	−0.03	−0.15	−0.22	0.10	−0.11
	P	0.00		0.85	0.29	0.12	0.50	0.46
AUCg cortisol	R	−0.03	−0.03	1	0.80^b^	0.20	0.05	−0.16
	P	0.85	0.85		0.00	0.15	0.76	0.27
AUCi cortisol	R	−0.16	−0.15	0.80^b^	1	0.04	−0.03	−0.13
	P	0.26	0.29	0.00		0.78	0.85	0.36
AI F4/F3	R	−0.13	−0.22	0.20	0.04	1	0.22	0.03
	P	0.35	0.12	0.15	0.78		0.13	0.84
AI F8/F7	R	−0.02	0.10	0.05	−0.03	0.22	1	0.30^a^
	P	0.87	0.50	0.76	0.85	0.13		0.03
AI O2/O1	R	−0.14	−0.11	−0.16	−0.13	0.03	0.30^a^	1
	P	0.35	0.46	0.27	0.36	0.84	0.03	

a significant at p < 0.05, uncorrected.

b significant at p < 0.01, uncorrected.

### EEG during rest after stress induction

#### Influence of stress on asymmetry

The asymmetry analysis conducted for the EEG measurements during the TSST and P-TSST condition was repeated also for the resting state data that were collected afterwards. Thus, we again performed a 2 x 3 repeated measures ANOVA with the factors condition (TSST, P-TSST) and electrode pair (F3/4, F7/8, O1/2) for AIs in alpha band power (see Table S2). There was neither a significant main effect of condition or electrode pair, nor a significant interaction between these factors (all *p*s>=0.109, see Table S3). Exploratory results for all electrode pairs can be found in Figure S3.

We calculated bivariate Pearson correlations between stress markers and AIs at the electrodes of interest during rest. None of the correlations were significant (see Table 2).

**Table 2:** Pearson correlations coefficients between stress markers and AIs during rest

		AUCg sAA	AUCi sAA	AUCg cortisol	AUCi cortisol	AI F4/F3	AI F8/F7	AI O2/O1
AUCg sAA	r	1,00	,36^a^	0,05	−0,07	0,02	0,17	−0,15
	*p*		0,01	0,74	0,63	0,89	0,23	0,30
AUCi sAA	r	,36^a^	1,00	0,02	0,21	−0,11	0,23	−0,03
	*p*	0,01		0,89	0,14	0,45	0,11	0,82
AUCg cortisol	r	0,05	0,02	1,00	−0.27	−0,03	−0,07	−0,07
	*p*	0,74	0,89		0,05	0,83	0,63	0,65
AUCi cortisol	r	−0,07	0,21	−0.27	1,00	0,24	0,03	0,12
	*p*	0,63	0,14	0,05		0,10	0,84	0,39
AI F4/F3	r	0,02	−0,11	−0,03	0,24	1,00	0,00	−0,16
	*p*	0,89	0,45	0,83	0,10		0,99	0,26
AI F8/F7	r	0,17	0,23	−0,07	0,03	0.00	1,00	−0,04
	*p*	0,23	0,11	0,63	0,84	0.99		0,81
AI O2/O1	r	−0,15	−0,03	−0,07	0,12	−0,16	−0.04	1,00
	*p*	0,30	0,82	0,65	0,39	0,26	0,81	

a significant at p < 0.05, uncorrected

### Individual alpha power

To investigate differences in EEG asymmetries at our electrodes of interest for individual alpha frequency (IAF), we repeated the analysis performed with standard alpha power with individual alpha power calculated according to (Klimesch, 1999; see supplementary analysis 1). We performed a 2 x 3 repeated measures ANOVA with the factors condition (TSST, P-TSST) and electrode pair (F3/4, F7/8, O1/2) for AIs in IAF. We found no significant main effect on either of the factors, but a significant interaction between condition and electrode (F_(2,100)_ = 4.35, *p* = 0.016, η_p_^2^ = 0.08; see Tables S5 and S6). However, this effect did not survive Bonferroni-correction in a post hoc test (all *p*s >0.066, see Table S7).

Again, we calculated a 2 x 3 repeated measures ANOVA with the factors condition (TSST, P-TSST) and electrode pair (F3/4. F7/8. O1/2) for AIs in IAF calculated according to Quaedflieg et al. (2015; see supplementary analysis 2). We found no significant main effect on either of the factors. The significant interaction between condition and electrode was at trend level (see Tables S8 and S9).

## Discussion

In the present study, we investigated the influence of acute stress on alpha asymmetries using continuous EEG during the TSST and P-TSST as well as during a resting state condition following the stress or control procedure. Stress induction using the TSST was successful as indicated by higher cortisol and sAA levels as well as an increase in subjective stress. An unexpected effect was observed in the cortisol results as the cortisol levels in the P-TSST session were higher at T_0_ compared to the TSST session. We can only speculate why this was the case. Possibly, participants who performed the TSST first had negative expectations for the second P-TSST session possibly leading to an anticipatory increase in cortisol. For participants that performed the P-TSST first, however, there was no negative expectation for the P-TSST and neither for the TSST because participants received no information about what would happen in the next session. However, because we did not find a general effect of order on subjective and endocrinological stress measures, this seems unlikely.

We found significantly higher AIs on the F3/F4 electrode pair in the stress compared to the placebo condition during stress. This represented stronger alpha power in the right hemisphere under stress at these frontal electrode pairs indicative of stronger left hemispheric activation as stronger alpha power is functionally linked to inactivation (Bazanova and Vernon, 2014). At the O1/O2 electrode pair, however, we found a significant difference but in the opposite direction with higher alpha power in the left hemisphere indicative of relatively stronger right-hemispheric activation. There were no differences between the TSST and P-TSST session in the following resting state EEG. Moreover, we repeated the analyses with AIs based on IAF. Following the methods of Klimesch, 1999, there was a significant interaction between condition and electrode, which did not survive Bonferroni-correction. Following the methods of Quaedflieg et al. (2015), there were no significant main effects or interactions (see supplementary analyses 1 and 2). Although the results using the methods of (Klimesch, 1999) are comparable to the results using standard alpha power, the results using the methods of Quaedflieg et al. (2015) do not agree. The interpretation of these differences, however, is limited by the fact that we did not measure individual alpha frequency during a closed eyes resting state before the start of the experiment. Thus, the discrepancies could result from these differences in methodology. Similar to the results of Quaedflieg et al. (2015), we only found changes on the F3/4 but not on the F7/8 electrode pair between the stress and control conditions. This contrasts findings from Zhang et al. (2018) who only found differences on the F7/8 but not the F3/4 electrode pair. Because these electrodes are adjacent to each other, it could be that the activity center lies between these electrodes and the discrepancies in the reported electrodes are because of small differences in cap positions of the EEG systems.

In line with the capability model of individual differences in asymmetry, we found significant differences in frontal alpha asymmetry during stress but not during the following resting state. This indicates that frontal asymmetries during emotional challenges are more pronounced compared to asymmetries during rest conditions. This idea is supported by the notion that the proportion of variance attributable to individual differences is higher during emotional challenge than at rest (Stewart et al., 2014). This possibly suggests that changes in alpha power during emotional challenge such as social stress could provide a more accurate predictor for the neurophysiological response to stress than alpha power asymmetries at rest.

The present results further indicate that the left frontal cortex was more active compared to the right under stress. This seems to be at odds with previous research on asymmetric cerebral activation of the hormonal stress response. Evidence from animal and human research points to an essential role of the right hemisphere in neuroendocrine and behavioral stress responses (Lueken et al., 2009): for example, the right ventromedial prefrontal cortex has been suggested to be dominant in the HPA-axis activation, whereas the medial PFC has been highlighted for incorporating stressful experiences (Sullivan and Gratton, 2002). Moreover, the right prefrontal cortex has been demonstrated to show increased activity in individuals with high stress responses and its activity is directly correlated with the cortisol response (Wang et al., 2005). Although the right frontal cortex seems to be more involved in the activation of the stress response (Sullivan and Dufresne, 2006), left hemispheric activation has been associated with the downregulation of the HPA-axis activation through interhemispheric inhibition (Sullivan, 2004). Thus, it is possible that the observed increase in left frontal activity could be attributed to emotion regulation processes during acute stress induction. Activity in the left frontal cortex is associated with reappraisal of negative emotional situations (Papousek et al., 2017). Our findings, therefore, fall in line with the asymmetric inhibition model of emotion regulation (Grimshaw and Carmel, 2014): according to this model, activation in the amygdala and ventral striatum focus the attention on salient emotional stimuli which leads to an appraisal in the orbitofrontal cortex. This in turn results in activation of the anterior insula giving rise to the subjective feeling. This activation cascade can be inhibited by the dorsolateral prefrontal cortex (dlPFC). However, this inhibition is asymmetric in nature as the left dlPFC inhibits negative distractors, whereas the right dlPFC inhibits positive distractors. As negative affect hinders performance in the TSST, these emotions could act as negative distractors (Henze et al., 2017).

As opposed to increased relative left hemispheric activation over frontal sites, we found increased right hemispheric activation over occipital sites. This result was unexpected. However, a potential explanation for this ostensible discrepancy concerns the coding for negative affect by the right hemisphere (Demaree et al., 2005). Although frontal areas have been hypothesized to code emotion differentially between the two hemispheres either because of valence (Sutton et al., 1997) or behavioral activation and inhibition (Harmon-Jones and Sigelman, 2001), posterior areas encode affect irrespective of valence in the right hemisphere (Killgore and Yurgelun-Todd, 2007; Packheiser et al., 2021). It could be speculated that although the changes in alpha asymmetry at anterior sites reflect regulation processes, the changes at occipital sites are related to the processing of the negative stressful situation. Thus, during the P-TSST, participants were in a neutral to positive mood reflected by more left-sided activity at posterior sites which becomes more right-sided activity under stress. Another possible explanation for this effect could be increased vigilance under stress (Henckens et al., 2016) and thus affect visual processing (Shackman et al., 2011) of environmental stimuli which displays a right hemispheric dominance in the visual cortex (Hellige et al., 2010). This could have led to stronger activation of the right visual cortex under stress. To investigate if changes at the O1/O2 electrode pair are related to the effects of stress induction at posterior sites, it would be interesting to look at different electrodes at parietal sites or to compute average asymmetry changes across several electrodes.

To use alpha asymmetries as predictors for psychopathology, behavior, or endocrinological responses, it has to be ensured that they provide a reliable measure as a variable can only be as correlated with another variable as with itself (Brysbaert, 2020). Although the reliability of alpha asymmetries within the same session is high (Koller-Schlaud et al., 2020), a recent study found that frontal alpha power asymmetries are not particularly reliable across several time points of measurement (Metzen et al., 2021) ranging from intraclass correlations of 0.5–0.6. In this study, time between recordings was on average 56.74 days. This does not necessarily mean that alpha power asymmetries are unreliable by themselves but rather depend on situational factors such as stress or mood changes from day to day. This conclusion is supported by our results as the present study demonstrates the state-dependency of alpha power asymmetries: although there was no change in alpha power asymmetry during rest after stress or nonstress, we could observe changes in alpha power asymmetries during the stressful situation itself. It should be noted that the reliability of individual alpha asymmetries has not been assessed in large samples and over long time periods. Individual alpha asymmetry has been suggested to be more suitable for the analysis of alpha oscillations during rest because alpha peak activity shows interindividual variability across subjects (Smulders et al., 2018). It could be that asymmetries of IAF thus show improved long-term reliability.

### Limitations of the study

One limitation of the current study is the method of individual alpha power determination. Typically, individual alpha power is centered on the peak frequency in the alpha power band for each participant during a closed eyes resting state before the start of the experiment. As we did not record a pretest resting state, we calculated IAF for each of the four EEG measurements (during TSST, during P-TSST, rest after TSST, and rest after P-TSST). Thus, we put the focus of this paper on standard alpha frequency. To an extent, this limits the comparability with previous works that use IAF.

Another limitation pertains to the sample selection. We only tested male participants to control for possible changes in hemispheric asymmetries in women because of cycle dependent fluctuations in sex hormones (Hausmann, 2017). As these hormones have been shown to influence hemispheric asymmetries, there could be a possible interaction in female participants. Moreover, sex differences in HPA reactivity and its influence on cognitive processes have been repeatedly reported (Merz and Wolf, 2017). It is yet to be seen if our results generalize to women.

A further potential confound could result from movement during data acquisition as participants had to actively perform during the TSST. However, previous research in our group has shown that asymmetries in alpha oscillations are unaffected by head movement likely because of movement affecting both hemispheres equally (Packheiser et al., 2020). Moreover, data quality in our study was high and did not differ between the two sessions. As EEG excels in temporal precision but lacks in spatial resolution, especially in subcortical structures, future studies should employ additional methods like fMRI. Further, the causal role of frontal asymmetries could be investigated via application of transcranial magnetic stimulation (Yadollahpour et al., 2019).

### Conclusion

In summary, we found that left frontal activity was increased during social stress but not during a following rest period supporting both the asymmetric inhibition as well as the capability model of emotional regulation. Over occipital sites, we found stronger right hemispheric activation during stress suggesting that negative affect may be dominantly processed in the posterior right hemisphere. Future research should focus on the association between stress and hemispheric asymmetries on a subcortical and network level and should explore the role of specific SNS and HPA contributions.

## STAR★Methods

### Key resource table

**Table:** 

REAGENT OR RESOURCE	SOURCE	IDENTIFIER
**Software and algorithms**		

Matlab 2018a	https://de.mathworks.com/products/matlab.html	RRID:SCR_001622
Presentation (Neurobehavioral Systems)	www.neurobs.com	RRID:SCR_002521
Corel Graphics Suite	https://www.coreldraw.com/	RRID:SCR_013674
IBM SPSS Statistics	https://www.ibm.com/products/spss-statistics	RRID:SCR_019096
BrainVision Recorder	https://www.brainproducts.com/	RRID:SCR_016331
BrainVision Analyzer	https://www.brainproducts.com/	RRID:SCR_002356

### Resource availability

#### Lead contact

Further information and requests for resources and reagents should be directed to and will be fulfilled by the lead contact, Gesa Berretz ( gesa.berretz@rub.de).

#### Materials availability

This study did not generate new unique reagents.

### Experimental model and subject details

#### Participants

51 male participants were recruited at the Ruhr University Bochum, Germany. We only included male participants as fluctuations in sex hormones in female participants can affect hemispheric asymmetries (Hausmann, 2017). All participants underwent stress and placebo sessions detailed in this manuscript followed by two experimental tasks assessing visual processing and lexical decision-making detailed elsewhere (Berretz et al., 2022). Handedness was assessed using the Edinburgh Handedness Inventory (Oldfield, 1971). Handedness was determined by calculating lateralization quotients (LQs) using the formula:

LQ = [(Right Preference – Left Preference) / (Right Preference + Left Preference)] ∗ 100

Eight participants were left-handed, as categorized by an LQ<0, and 43 were right-handed with an LQ>0 (sample mean=64.29, SD=62.09). Following recent recommendations for neuroscience studies on hemispheric asymmetries (Willems et al., 2014) we did not exclude left-handers in order to get a more representative sample of the actual distribution of hemispheric asymmetries in the population.

Participants were aged between 18 and 39 years (M=24.5, SD=5.04). All participants were healthy with no history of mental or neurological disorders; all were non-smokers and had no prior experience with the stress paradigm. To control for possible influences on the cortisol response during the experiment, all participants had a body mass index between 18.5-25 kg/m^2^, took no medication, took no drugs and were not performing shiftwork (Herhaus and Petrowski, 2018; Kirschbaum et al., 1995; Labuschagne et al., 2019). The local ethics committee of the Faculty of Psychology at the Ruhr University Bochum approved the study. This experiment was part of a larger study investigating the influence of stress on hemispheric asymmetries. All participants were treated in accordance with the Declaration of Helsinki and gave written informed consent. Participants received a compensation of 50€ or course credit.

### Method details

#### Procedure

Participants were invited for two test sessions. Sessions took place between 2-6 pm to control for circadian changes in cortisol (Labuschagne et al., 2019). The general experimental design is shown in Figure S1. After providing written informed consent, participants were setup with the EEG cap and were instructed to minimize their head and facial movements. All participants completed baseline subjective stress and cortisol measurements, after which the stress induction or a control procedure were applied. Subjective stress was assessed with the Subjective Experiences Rating Scale (SERS; Kahan and Claudatos, 2016) as well as a set of visual analog scales that measure subjective perception of stress (VAS; Kudielka et al., 2004). For stress induction, we used the Trier Social Stress Test (TSST) (Kirschbaum et al., 1993). After a five-minute preparation period, participants had to give a five-minute oral presentation about their positive traits in a mock job interview followed by a mental arithmetic task (subtracting in steps of 17) for a total of 10 minutes. During the presentation and the arithmetic task, a panel consisting of a woman and a man dressed in lab coats evaluated the participants. The panel acted very reserved and refrained from giving any positive feedback. Furthermore, the participant’s face was being videotaped and the video was streamed to a nearby monitor allowing participants to view their own performance. The panel consisted of trained student assistants who followed the same stress induction routine for every participant. Neither the panel nor the participants were aware of the hypothesis of the experiment. In addition to pointing out mistakes in the participant’s performance, the panel also urged participants to keep their head still to ensure high EEG data quality.

As a control condition, we utilized the Placebo-TSST (P-TSST, Het et al., 2009). It also consisted of a preparation period, an oral presentation and an arithmetic task. However, participants were neither monitored nor filmed, and the mental arithmetic task was less taxing (counting forward in steps of 15). After the preparation period, participants were informed to start talking about their last vacation and when to start counting. For each task, the experimenter left the room. The P-TSST lacks the stressful elements of the TSST like social evaluation and pressure to perform (Dickerson and Kemeny, 2004) while mimicking its task demands. Therefore, it is a suitable control procedure. As the participants were alone during the P-TSST, they were only reminded to keep their head still at the beginning of recording.

The order of TSST and P-TSST sessions were pseudo-randomized so that half the participants began with the TSST session and the other half with the P-TSST session. Following the stress induction or the placebo condition, 5 minutes of eye-closed resting state EEG were recorded. Salivary samples were collected using Salivette sampling devices (Sarstedt, Nümbrecht, Germany). The first sample was collected as baseline before the stress induction. The second sample was collected after stress induction before the resting state EEG was measured. The three following samples were collected at an interval of approximately 15 minutes.

#### EEG recording and analysis

EEG data during stress induction and control condition were recorded using a 64 Ag–Ag Cl electrode system (actiCAP ControlBox and QuickAmp, Brain Products GmbH, Gilching, Germany), positioned at standard scalp locations according to the International 10–20 system (FCz, FP1, FP2, F7, F3, F4, F8, FC5, FC1, FC2, FC6, T7, C3, Cz, C4, T8, TP9, CP5, CP1, CP2, CP6, TP10, P7, P3, Pz, P4, P8, PO9, O1, Oz, O2, PO10, AF7, AF3, AF4, AF8, F5, F1, F2, F6, FT9, FT7, FC3, FC4, FT8, FT10, C5, C1, C2, C6, TP7, CP3, CPz, CP4, TP8, P5, P1, P2, P6, PO7, PO3, POz, PO4, PO8). Data were recorded with a sampling rate of 1000 Hz. The FCz electrode was used as reference during recording, but later re-referenced (see below). Impedances were kept under 5kΩ at the beginning of recording.

Data analysis was performed using the Brain Vision Analyzer software (Brain Products GmbH) following the procedure previously used by Ocklenburg et al. (2019). First, visual data inspection to reject EEG-sections containing technical artifacts and exclusion of faulty or flatlined channels was performed. After that, a semiautomatic independent component analysis (ICA) with Infomax rotation was applied to eliminate reoccurring artifacts like pulse, blinks and eye movements. Next, the FCz and missing or rejected channels were interpolated using topographical interpolation with spherical splines. The first and last 30 seconds of recording were discarded. A band pass filter with a low cutoff of 1 Hz and a high cutoff of 30 Hz was applied. Data were segmented into intervals of 1024ms skipping bad intervals. Segments were baseline corrected over the complete segments to eliminate drift and an automatic artifact rejection was performed. For the automatic artifact rejection, segments were excluded if voltage steps of 50 μV/ms, value differences of more than 200 μV within a 200 ms interval or signal strengths below 0.5 μV within a 100ms interval occurred. Subsequently, a CSD-transformation (Peters and Servos, 1989) was applied in order to eliminate the reference potential from the data. Finally, we performed a fast Fourier transformation with a 10% Hamming window in accordance with the Brain Vision Analyzer User Manual and averaged data across segments. For statistical analysis, we extracted alpha band power (8-12 Hz).

#### Endocrinological measurements

Saliva analyses were conducted in the in-house laboratory of the Departments of Genetic Psychology and Cognitive Psychology at the Ruhr University Bochum. Saliva was analyzed using a cortisol enzyme-linked immunosorbent assay (Cortisol Saliva ELISA, IBL, Hamburg, Germany) with intra-assay coefficients of variance (CV) below 5% and inter-assay CVs below 15%.

In addition, the enzyme alpha-amylase (sAA) was analyzed from the saliva samples for assessing the response of the sympathetic nervous system (Rohleder and Nater, 2009). A colorimetric test using 2-chloro-4-nitrophenyl-α-maltrotriosoide (CNP-G3) as a substrate reagent was applied to measure sAA concentration (Lorentz et al., 1999; Winn-Deen et al., 1988). Intra- and inter-assay variabilities were below 10%.

### Quantification and statistical analysis

#### Statistical analysis

Statistical analysis was performed using SPSS software version 20 (IBM). For cortisol and sAA, we calculated the area under the curve with respect to increase (AUC_i_) following the formula described by Pruessner et al. (2003). To check the stress manipulation, we calculated a repeated measures ANOVA between the factor condition (two levels: TSST and P-TSST) and the factor time points of measurement (five levels: T_0_, T_15_, T_20_, T_35_ and T_50_). For the recorded EEG signal, we calculated asymmetry indices (AIs) for the alpha power for each electrode pair following the formula by Reznik and Allen (2018):

AI = ln(right) − ln(left)

Positive AI values indicate stronger power on the right side, while negative AI values indicate stronger power on the left side. In our main analysis, we focused on asymmetries scored from the electrode pairs F3/4, F7/8 and O1/2. The frontal electrodes were chosen as previous research by Quaedflieg et al. (2015) focused on these electrode positions. The occipital electrodes were chosen because they map onto the alpha frequency generators in the brain (Bazanova and Vernon, 2014). To evaluate the influence of stress on frontal asymmetries, we performed a repeated measures ANOVA with the factors condition and electrode pair. All post-hoc tests were Bonferroni corrected to control for multiple comparisons. All following analyses were calculated with the full sample of 51 participants.

## Data Availability

No data or original code has been deposited online due to ethical constraints. Any information required to reanalyze the data reported in this paper is available from the lead contact upon request.
