# Supplementary material for: Acute stress increases left hemispheric activity measured via changes in frontal alpha asymmetries
Source: iScience. 2022 Feb 1;25(2):103841. doi: 10.1016/j.isci.2022.103841 (PMC8850739; doi:10.1016/j.isci.2022.103841)
Supplement: Document S1. Figures S1–S3, Data S1–S4, and Tables S1–S9 [file mmc1.pdf]

iScience, Volume *25*

Supplemental information

Acute stress increases left hemispheric activity measured via changes in frontal alpha asymmetries

Gesa Berretz, Julian Packheiser, Oliver T. Wolf, and Sebastian Ocklenburg

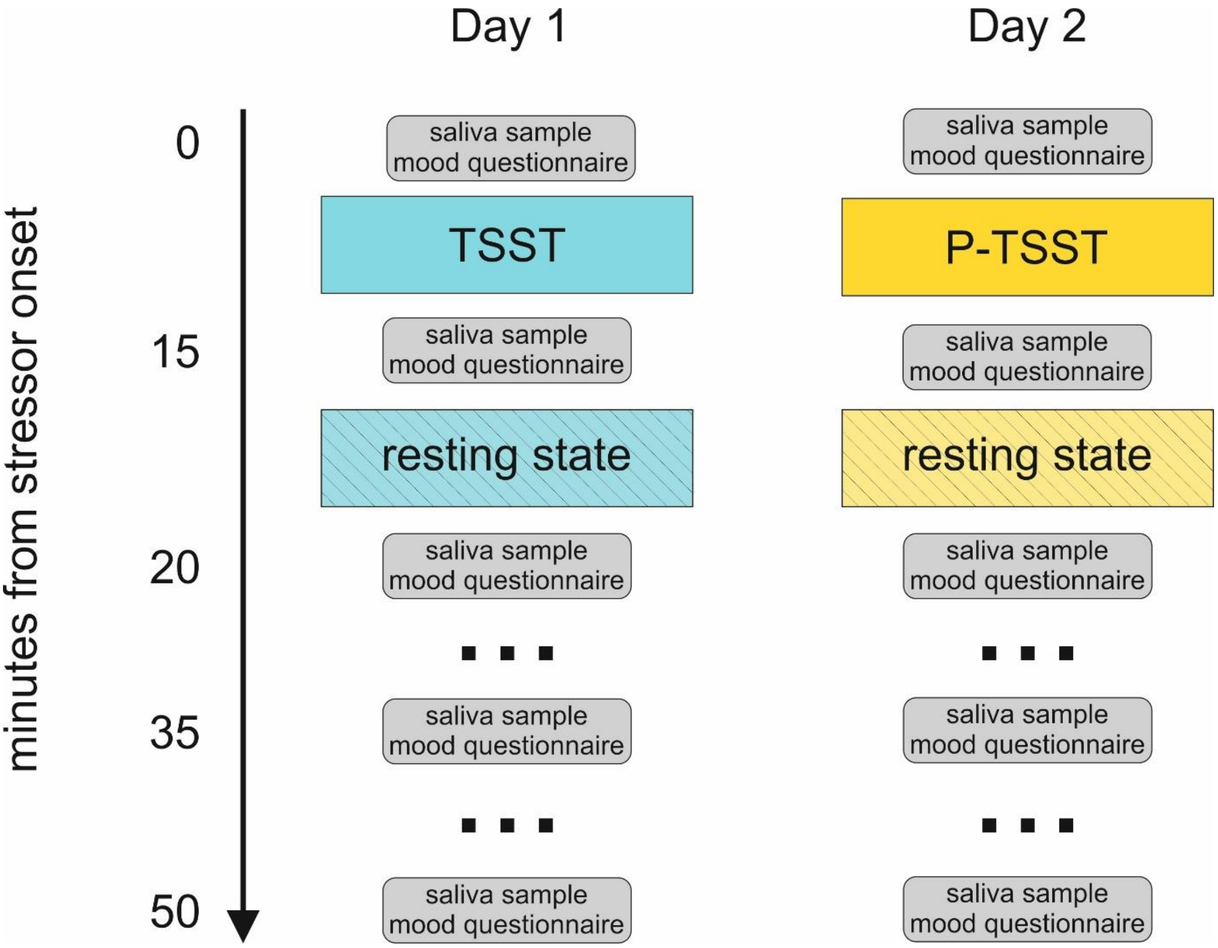

Supplementary Figure S1 (related to STAR Methods). Experimental design. After TSST or P-TSST, the participants complete a resting state EEG followed by two tasks that are not content of this study. Before the stress induction and after each section of the experiment, cortisol, sAA and affect are assessed. Participants wear an EEG throughout the whole experiment. Order of day one and two were randomized.

Supplemental Data S1 (related to Figure 2). Asymmetry during stress

We performed a 2 x 28 repeated measures ANOVA with the factors condition (TSST, P-TSST) and electrode pair (FP1/2, AF3/4, AF7/8, F1/2, F3/4, F5/6, F7/8, FC1/2, FC3/4, FC 5/6, FT7/8, FT9/10, C1/2, C3/4, C5/6, T7/8, CO1/2, CP3/4, CP5/6, TP7/8, TP9/10, P1/2, P3/4, P5/6, P7/8, PO3/4, PO7/8, O1/2) for AIs in alpha band power. There was a significant main effect of electrode ($F_{(72,1350)}$=2.12, p<.001, η_p^2=.04). Moreover, there was a significant interaction between condition and electrode ($F_{(27,1350)}$=1,51, p=.047, η_p^2=.03). Bonferroni-corrected post-hoc tests revealed a significantly higher AI at the F3/4, F5/6, TP9/10 electrode pair in the TSST condition compared to the P-TSST condition (all *p*s<.048). At the FT9/10 and O1/2 electrode pair, this effect was reversed with lower AI scores in the TSST condition (all *p*s<.028).

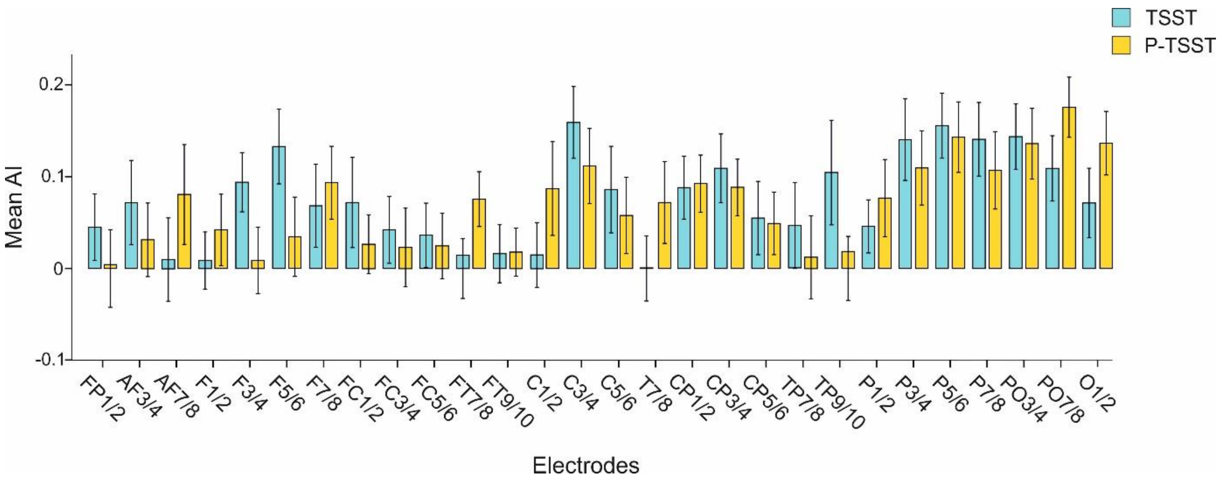

Supplementary Figure S2 (related to Figure 2). Alpha asymmetry indices during stress (TSST) and no stress (P-TSST) for all electrodes. Error bars represent ±1 SEM.

Supplemental Data S2 (related to Figure 2). Asymmetry during rest after stress

We performed a 2 x 28 repeated measures ANOVA with the factors condition (TSST, P-TSST) and electrode pair (FP1/2, AF3/4, AF7/8, F1/2, F3/4, F5/6, F7/8, FC1/2, FC3/4, FC 5/6, FT7/8, FT9/10, C1/2, C3/4, C5/6, T7/8, CO1/2, CP3/4, CP5/6, TP7/8, TP9/10, P1/2, P3/4, P5/6, P7/8, PO3/4, PO7/8, O1/2) for AIs in alpha band power. There was a significant main effect of electrode ($F_{(72,1350)}$=5.46, p<.001, η_p^2=.1). There were no other significant main effects or interactions.

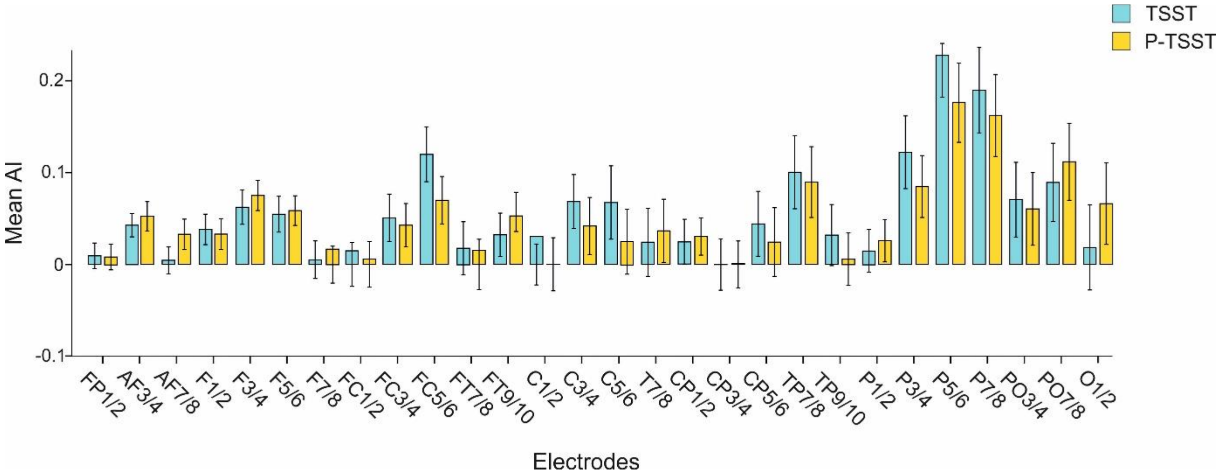

Supplementary Figure S3 (related to Figure 2). Alpha asymmetry indices during rest after stress (TSST) and no stress (P-TSST) for all electrodes. Error bars represent ±1 SEM.

Supplementary Table S1 (related to Figure 1). Descriptive data of stress markers.

Stress marker	Condition	Measurement time point	N	Min	Max	Mean	Standard deviation
Cortisol	TSST	T_0	51	.79	14.48	3.89	2.52
		T_{15}	51	.00	16.11	5.43	3.73
		T_{20}	51	2.00	26.83	8.76	5.60
		T_{35}	51	.00	21.51	9.48	4.85
		T_{50}	51	1.63	24.72	8.27	4.60
	P-TSST	T_0	51	1.33	30.52	6.78	4.82
		T_{15}	51	1.01	17.14	4.95	3.45
		T_{20}	51	.00	13.96	4.29	2.75
		T_{35}	51	.83	9.98	3.78	2.02
		T_{50}	51	.75	8.99	3.28	1.78
sAA	TSST	T_0	51	31.75	475.86	191.04	89.57
		T_{15}	51	46.38	989.51	309.06	194.96
		T_{20}	51	31.98	495.48	203.10	114.87
		T_{35}	51	44.58	601.81	207.05	121.23
		T_{50}	51	29.13	551.63	197.83	114.50
	P-TSST	T_0	51	26.17	703.95	216.84	138.48
		T_{15}	51	20.04	755.45	222.48	155.94
		T_{20}	51	15.12	540.81	184.68	122.64
		T_{35}	51	19.75	480.98	197.00	119.01
		T_{50}	51	13.55	606.93	203.33	122.82
SERS	TSST	T_0	51	15.00	32.00	19.76	3.98
		T_{15}	51	15.00	54.00	31.43	9.17
		T_{20}	51	15.00	42.00	21.47	6.11
		T_{35}	51	15.00	35.00	21.78	5.43
		T_{50}	51	15.00	34.00	21.04	4.45
	P-TSST	T_0	51	15.00	34.00	20.12	3.95
		T_{15}	51	15.00	42.00	23.31	6.35
		T_{20}	51	15.00	39.00	19.12	5.20
		T_{35}	51	15.00	38.00	22.51	4.94
		T_{50}	51	15.00	47.00	23.22	6.55

Supplementary Table S2 (related to Figure 2). Descriptive data of AIs.

			Mean	SD	N
During stress	TSST	F3/F4	0.08	0.19	51
		F7/F8	0.06	0.23	51
		O1/O2	0.06	0.27	51
	P-TSST	F3/F4	0.01	0.22	51
		F7/F8	0.12	0.21	51
		O1/O2	0.08	0.24	51
During rest	TSST	F3/F4	0.06	0.13	51
		F7/F8	0.01	0.14	51
		O1/O2	0.02	0.32	51
	P-TSST	F3/F4	0.07	0.12	51
		F7/F8	-0.02	0.14	51
		O1/O2	0.07	0.31	51

Supplementary Table S3 (related to Figure 2). ANOVA of AIs.

		df	F	p	Partial η^2
During stress	Condition	1	0.02	0.90	0.00
	Electrodes	2	0.81	0.45	0.02
	Condition * Electrodes	2	3.36	0.04	0.06
During rest	Condition	1	1.57	0.22	0.03
	Electrodes	2	2.07	0.13	0.04
	Condition * Electrodes	2	2.26	0.11	0.04

Supplementary Table S4 (related to Figure 2). Descriptive data of power of electrodes of interest.

Electrode	N	Min	Max	Mean	Standard deviation
F3 TSST	51	12.01	44.08	19.05	5.51
F3 P-TSST	51	11.00	43.59	19.90	5.62
F4 TSST	51	9.70	41.42	20.81	6.62
F4 P-TSST	51	11.57	31.46	19.82	4.13
O1 TSST	51	14.83	53.87	27.04	9.65
O1 P-TSST	51	14.90	80.22	28.26	12.30
O2 TSST	51	14.07	82.68	28.68	11.02
O2 P-TSST	51	16.66	84.90	31.95	14.47
F7 TSST	51	14.18	71.96	28.27	9.90
F7 P-TSST	51	15.09	44.95	25.99	6.22
F8 TSST	51	15.57	56.51	29.78	9.28
F8 P-TSST	51	17.62	51.09	28.46	8.42

Supplemental Data S3 (related to Figure 2): Individual alpha frequency according to Klimesch et al. (1999)

We calculated IAF for each of the four EEG measurements (during TSST, during PTSST, rest after TSST, rest after PTSST). We used the individual peak alpha frequency within each condition to approximate the individual alpha frequency. Following the methods detailed in Klimesch et al. (1999) the individual alpha band was calculated as follows:

Lower bound: individual peak frequency – 4Hz

Upper bound: individual peak frequency + 2Hz (see supplementary table 5)

Supplementary Table S5 (related to Figure 2). Descriptive data of AIs of IAF (Klimesch et al., 1999).

			Mean	SD	N
During stress	TSST	F3/F4	0.09	0.18	50
		F7/F8	0.00	0.26	50
		O1/O2	0.05	0.24	50
	P-TSST	F3/F4	0.02	0.23	50
		F7/F8	0.08	0.24	50
		O1/O2	0.11	0.22	50
During rest	TSST	F3/F4	0.06	0.12	51
		F7/F8	0.00	0.14	51
		O1/O2	0.02	0.30	51
	P-TSST	F3/F4	0.07	0.11	51
		F7/F8	-0.01	0.14	51
		O1/O2	0.06	0.30	51

Supplementary Table S6 (related to Figure 2). ANOVA of AIs of IAF (Klimesch et al., 1999).

		df	F	p	Partial η^2
During stress	Condition	1	1.05	0.31	0.02
	Electrodes	2	0.71	0.50	0.01
	Condition * Electrodes	2	4.35	0.02	0.08
During rest	Condition	1	2.01	0.16	0.04
	Electrodes	2	2.15	0.12	0.04
	Condition * Electrodes	2	1.83	0.17	0.04

Supplementary Table S7 (related to Figure 2). Post-hoc test.

Pairwise comparison							
Electrode	(I) Condition	(J) Condition	Mean difference (I-J)	Standard error	p	95% confidence interval for the mean difference	
						lower	upper
F3/F4	1	2	0.07	0.04	0.08	-0.01	0.15
	2	1	-0.07	0.04	0.08	-0.15	0.01
F7/F8	1	2	-0.08	0.04	0.09	-0.16	0.01
	2	1	0.08	0.04	0.09	-0.01	0.16
O1/O2	1	2	-0.06	0.03	0.07	-0.13	0.00
	2	1	0.06	0.03	0.07	0.00	0.13

Supplemental Data S4 (related to Figure 2): Individual alpha frequency according to Quaedflieg et al. (2015)

We calculated IAF for each of the four EEG measurements (during TSST, during P-TSST, rest after TSST, rest after PTSST) following the methods detailed in Klimesch et al. (1999). The individual alpha band was calculated as follows:

IAF = individual peak frequency +- 0.2 x individual peak frequency (see supplementary table 8)

Supplementary Table S8 (related to Figure 2). Descriptive data of AIs of IAF (Quaedflieg et al., 2015).

			Mean	SD	N
During stress	TSST	F3/F4	0.08	0.18	50
		F7/F8	0.01	0.26	50
		O1/O2	0.06	0.24	50
	P-TSST	F3/F4	0.02	0.23	50
		F7/F8	0.08	0.26	50
		O1/O2	0.11	0.22	50
During rest	TSST	F3/F4	0.06	0.13	51
		F7/F8	0.01	0.15	51
		O1/O2	0.02	0.33	51
	P-TSST	F3/F4	0.07	0.12	51
		F7/F8	-0.02	0.15	51
		O1/O2	0.08	0.33	51

Supplementary Table S9 (related to Figure 2). ANOVA of AIs of IAF (Quaedflieg et al., 2015).

		df	F	p	Partial η^2
During stress	Condition	1	0.68	0.41	0.01
	Electrodes	2	0.705	0.49	0.01
	Condition * Electrodes	2	3.019	0.05	0.06
During rest	Condition	1	1.238	0.27	0.02
	Electrodes	2	2.027	0.14	0.04
	Condition * Electrodes	2	2.73	0.07	0.05
